# Electromagnetic exposure levels of electric vehicle drive motors to cochlear implanted passenger

**DOI:** 10.1371/journal.pone.0322735

**Published:** 2025-05-14

**Authors:** Xu-Wei Dong, Yi-Dan Qian, Mai Lu

**Affiliations:** Key Laboratory of Opto-Electronic Technology and Intelligent Control of Ministry of Education, Lanzhou Jiaotong University, Lanzhou, China; UFPE: Universidade Federal de Pernambuco, BRAZIL

## Abstract

In order to evaluate the effects of electromagnetic radiation generated by the dual-drive motors of an electric vehicle on special passengers with cochlear implanted, this study considers a cochlear implanted passenger as the research object, takes the drive motors in electric vehicle as the exposure source. A calculation model including the vehicle body, brain tissue, skull, eyes, human body, and cochlear implant is built, and the finite element method is used to calculate the induced electric field ($E_{in}$), specific absorption rate (SAR), and temperature changes in different tissues and organs of the passenger’s body. The results show that the maximum value of $E_{in}$ on the human body surface is 60.8 mV/m at the ankle. The $E_{in}$ around the cochlear implant inside the human head is also high, with a maximum value of 57.1 mV/m. The maximum SAR of the human body is $\;1.99\times 10^{-6}\;W/kg$, which also appears near the cochlear implant. Besides, the maximum temperature rise of the human body, brain tissue, and cochlear implant is 0.10 °C, 0.28 °C, and 0.0076 °C, respectively. Calculation shows that the $E_{in}\;$ and SAR of the human body and different tissues are much lower than the safety limit specified in the guidelines of the International Commission on Non-Ionizing Radiation Protection (ICNIRP), and the temperature rise does not reach the thermal damage threshold in the guidelines. The electric field around the electrode tip and the surface of the cochlear implant, the temperature rise of the cochlear implant also meet the requirements of the ICNIRP and the International Organization for Standardization’s 14708–7 medical device standard. The results could enrich the study on the electromagnetic environment of electric vehicles and provide references for the design and improvement of cochlear implants and electromagnetic exposure protection for vehicles.

## Introduction

As an important tool to promote the transition to low-carbon road transport [1], electric vehicles (EVs) play a crucial role in addressing the challenge of global climate change and reducing the dependence on fossil fuel. These vehicles provide an environmentally friendly and sustainable mobility solution and substantially contributes to greenhouse gas reduction and air quality improvement. According to the International Energy Agency [2], global ownership of EVs will increase to 70 million by 2025, and EVs are expected to account for 30% of the total transportation market share by 2030. However, with the increasing complexity of the electrical equipment and systems in EVs, electromagnetic compatibility and electromagnetic safety problems are becoming increasingly prominent and have become the focus of widespread attention. Therefore, scholars have conducted extensive research on the electromagnetic environment of EVs to ensure that EVs can serve the society stably and safely while developing rapidly and build a green, low-carbon transportation system as soon as possible.

Given the integration of many high-power electrical equipment inside EVs, the electromagnetic environment inside these vehicles is complex during driving. Tell et al. [3] measured the electromagnetic field between 120 Hz–10 kHz and 1.2–100 kHz in the carriage of electric and fuel vehicles during driving and found that the electric field ranges of electric and fuel vehicles are roughly the same, but the average magnetic field intensity of EVs is relatively high. Nevertheless, the electromagnetic field intensity measured in EVs is still within the limits specified by the ICNIRP. Pääkkönen and Korpinen [4] compared the magnetic fields near passenger seats inside electric, gasoline, and hybrid vehicles and discovered that the magnetic fields in gasoline and hybrid vehicles have approximately the same values, but the magnetic field in EVs is slightly lower. The measured results do not exceed the safety limit. Gombarska et al. [5] measured the electric field intensity generated by various communication and high-frequency electronic devices inside an EV within the frequency range of 500–3,300 MHz, and the results showed that the electromagnetic radiation inside the vehicle is also within the safe range. In addition, to reduce the effect of the electromagnetic field on the passenger’s body, researchers have studied the electromagnetic radiation shielding property of the vehicle body material [6–8].

In general, measuring the electromagnetic field in human living tissue via experimental measurement is impossible because of medical ethics. Many researchers have studied the possible effects of the electromagnetic environment generated by EVs on human body. Lin et al. [9] investigated the magnetic flux density at the rear seat position of an EV under an extremely low-frequency magnetic field and calculated the induced electric field in adults and children; they found that the induced electric field in children is much lower than that in adults. Tan et al. [10] established an equivalent electromagnetic model of the EV body and the human body, studied the distribution of the induced electric field in the body of a passenger exposed to radiation of the power cable. The results showed that the low-frequency magnetic field during the accelerating state of the EV considerably affects the human body. Shang et al. [11] studied the influence of high-frequency electromagnetic radiation from GPS antennas of EVs; calculated the induced electric field, specific absorption rate, and temperature changes of the human body at different positions within the vehicle after 30 min of exposure to GPS antenna radiation; and compared them with the values in ICNIRP’s 2020 guidelines. With the continuous development of wireless power technology for EVs, to ensure the safety and reliability of this technology, researchers [12–14] have analyzed the electromagnetic radiation of wireless charging systems with different power and compared the results with the ICNIRP’s guidelines [15,16] to assess the effects of electromagnetic radiation generated by the wireless charging process of EVs on human health.

While the electromagnetic fields in EVs are generally within safety limits for ordinary passengers, individuals with medical implants may face unique risks. In particular, electromagnetic interference may affect the function of medical implants with high electromagnetic sensitivity, thus affecting the life safety of patients. To ensure the safe travel and life safety of people wearing medical implants, many scholars have conducted relevant studies [17–20]. Although studies have initially revealed the effects of different electromagnetic environments on different medical implants, research on electromagnetic exposure of people wearing cochlear implants remains scarce. With the continuous popularity of EVs, the probability of cochlear implant wearers taking vehicles is also increasing. Conducting the study on electromagnetic exposure of cochlear implant wearers is not only related to personal safety, but also an important part in promoting the coordinated development of EVs and implantable medical devices and improving the relevant standard system.

According to the first global hearing report [21] released by the World Health Organization on March 2021, nearly 2.5 billion people (one quarter of the world’s population) will have some degree of hearing loss by 2050. Cochlear implant is the main means of hearing compensation and reconstruction for people with severe audiosensory deafness. However, because the electrode array in the implant contains metal materials, the electric field distribution in the cochlea tissues may substantially change if the electrode array is exposed to external electromagnetic radiation [22]. These changes may increase the intensity of energy absorption in the area adjacent to the implant, thus triggering local temperature rises, which may cause damage to surrounding human tissues. In the long term, this may induce abnormal stimulation of the auditory nerve and functional problems with the implanted device. Sibella et al. [23] considered the influence of the radiofrequency (RF) field during mobile phone calls and investigated whether cochlear implants have an effect on the specific absorption distribution in the human head. The results showed that the implant system meets the safety limits of typical exposure to 900 or 1,800 MHz mobile phones. Zeng et al. [24] used the transfer function method to evaluate the heating of cochlear implant induced by an MRI RF electromagnetic field and identified the factors that considerably influence such heating. Benova et al. [25] analyzed the passengers wearing cochlear implant exposed to an RF field for a long time due to the use of mobile phones during railway travel. The results suggested that prolonged use of mobile phones (900 MHz) in an enclosed space may be dangerous to people with cochlear implant.

Despite the widespread use of medical implants, only a few studies have examined the influence of electromagnetic field exposure to implanted wearers, especially the effects of electromagnetic fields in EVs on this type of passengers. Since the cochlear implant is sensitive to external electromagnetic fields, quantitative study can analyze the effects of external electromagnetic fields on the wearer so that appropriate measures can be implemented to protect the safety of patients and reduce the risk of potential adverse physiological reactions. Quantitative study can also complement research on the complex electromagnetic environment of EVs. Therefore, this study aims to quantitatively evaluate whether the high-frequency electromagnetic field generated by dual-drive motors in EVs will affects the passenger wearing cochlear implant. With the finite element analysis software Comsol Muitiphysics 6.0, dual-drive motors are used as electromagnetic radiation sources in EVs, and the distribution of the induced electric field, SAR, and temperature rise in the human body, brain, and other tissues of a passenger wearing a cochlear implant are calculated.

## Theory and method

As frequency increases, biological tissues absorb more electromagnetic waves, reducing penetration depth. In high-frequency ranges, such as microwaves and infrared, electromagnetic waves primarily propagate via radiation and are readily absorbed. These high-frequency electromagnetic waves can produce thermal effects inside tissues, thus affecting the physiological function of tissues.

SAR measures the electromagnetic energy absorbed by a substance per unit mass over time. It is often used to describe the absorption properties of a substance to a specific frequency of electromagnetic radiation. SAR is calculated as follows:


$$SAR=\frac{\sigma |\;E|\;2}{\rho }$$ (7)

where σ is electrical conductivity in S/m and *E* is the induced electric field in V/m.

When electromagnetic waves propagate inside human tissues, part of the energy is absorbed by the tissues and converted into heat energy, resulting in an increase in tissue temperature. To evaluate whether the thermal effect produced by high-frequency electromagnetic radiation influences the human body, this study uses the bioheat transfer module in Comsol to calculate the temperature rise of human tissues. In the calculation process, Pennes’ transient biological heat equation [26] is employed to analyze the temperature field distribution in human tissues. The bioheat transfer equation is as follows:


$$q=-k\frac{dT}{d\tau }$$ (8)

$$\rho c_{p}\frac{\partial T}{\partial t}+\nabla \cdot \left(q\right)=\rho \cdot SAR+\rho_{b}c_{p}w_{b}\left(T_{b}-dT\right)+Q_{met}$$ (9)

where ρ  refers to material density in $kg/m^{3}$, k is the thermal conductivity of the material in W/m·K,${\;w}_{b}\;$ is the blood perfusion rate in 1/s,${\;c}_{p}\;$ is the specific heat capacity of blood in J/kg·K,${\;T}_{b}\;$ is the temperature of blood in K, and $Q_{met}$ is a metabolic heat source in J/$m^{3}\cdot s$.

## Materials and models

### Finite element mesh of calculation model

**Fig 1:**
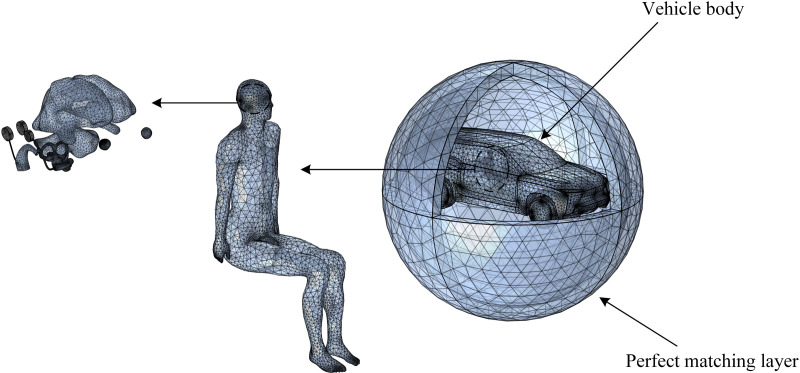
Meshing grids of the finite element model.

This study establishes a vehicle body model with the real scale. The length, width, and height of the vehicle body model are 5,099 mm × 1,989 mm × 1,750 mm. The body material is aluminum alloy. Only the outer contour of the vehicle is retained in the model to ensure the accuracy of the calculation results and reduce the complexity of finite element division. Fig 1 shows the tetrahedral meshing of the finite element model. The magnetoelectric insulated spherical outer boundary is used to simulate the free space at the edge of the domain. In this setting, the electromagnetic insulation condition is considered to be fully achieved because the electromagnetic wave propagating on the boundary is much smaller than that in the center of the calculated region.

### Equivalent electromagnetic model of driving motors

The driving motor is the core component of the EV power system, and the current inside the motor generates an electromagnetic field in the surrounding space and affects the environment in the form of electromagnetic radiation. Therefore, in this study, two drive motors in four-wheel-drive EVs are used as radiation sources. Given the complexity of the physical structure and circuit composition of the drive motor, if the model is completely based on the theoretical model of the motor, the model will become extremely complicated, the complexity of the finite element grid will greatly increase, abundant computing resources will be consumed, and non-negligible calculation errors may arise. Therefore, on the basis of the antenna radiation principle [27,28], the complex circuit structure of the driving motor is set to be equivalent to the dipole antenna model in this study.

The radiation characteristics of the dipole antenna is mainly depended on its length and operating frequency, the length of the antenna is set to half the wavelength of the electromagnetic wave in this study, that is, 3.75 m. The dipole arm length is set to 1/4 of the wavelength to ensure optimal resonant conditions. Moreover, the radius of the dipole arm is set to 1/20 of the wavelength to enhance structural stability and radiation efficiency. The gap between the two arms is set to 1/100 of the wavelength to reduce interference and promote the concentrated radiation of electromagnetic waves. A 0.3 V excitation source is applied to the dipole antenna to simulate the spatial electromagnetic field generated by the drive motor during operation through the induced electromagnetic field generated by the adjacent conductive surface.

**Fig 2:**
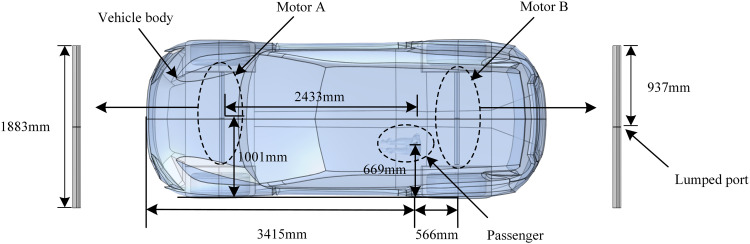
Electromagnetic environment model of EVs.

Some researchers have used the method of establishing an equivalent source to calculate and analyze the drive motor and measured the spatial electromagnetic field generated by the drive motor in the EV. The results showed that the spatial electromagnetic field reaches its maximum near 80 MHz when the drive motor runs at a speed of 6,000 r/min [29,30]. Therefore, in this study, an equivalent source model of the dipole antenna is constructed in Comsol Multiphysics software to study the effect of electromagnetic fields generated by the drive motors of EVs on passenger with cochlear implant, and the distribution of different induction fields in the body of passengers is calculated. The electromagnetic environment model of EVs is shown in Fig 2.

## Model of a passenger with a cochlear implant

### Cochlear implant model

**Fig 3:**
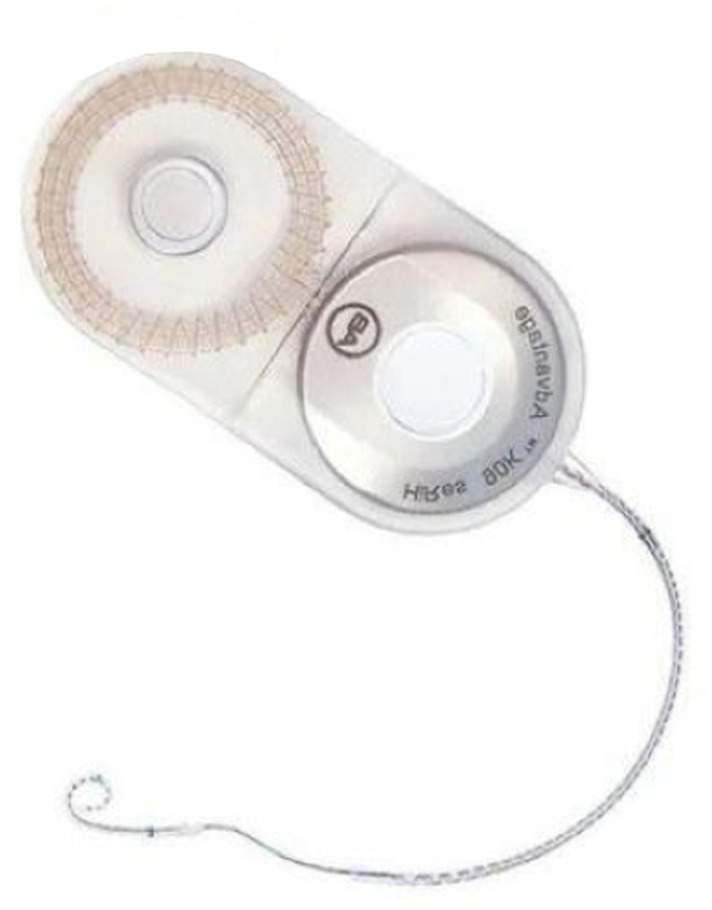
Physical structure of the cochlear implant.

**Fig 4:**
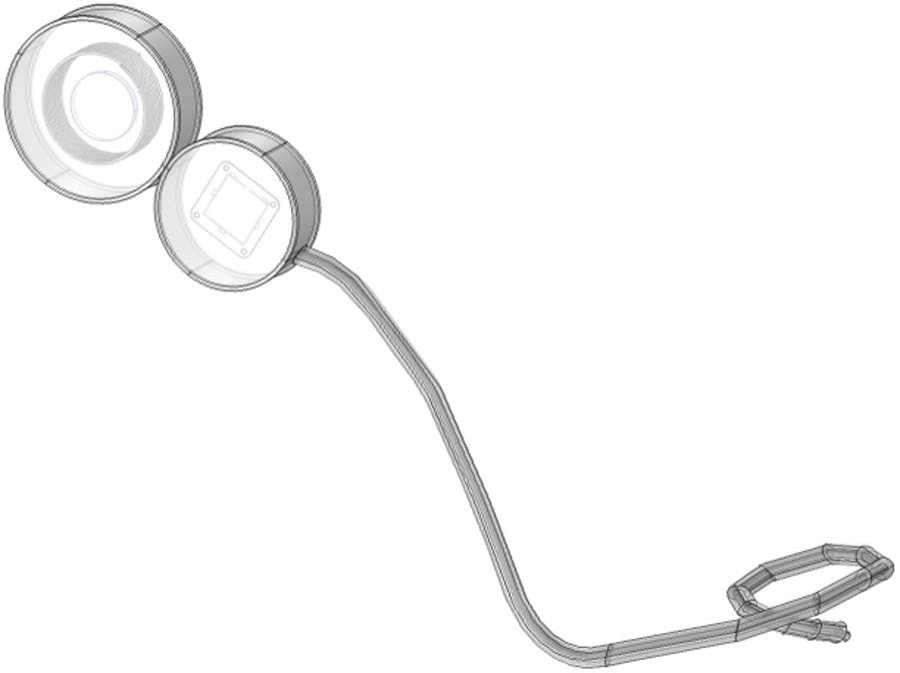
The cochlear implant model.

**Fig 5:**
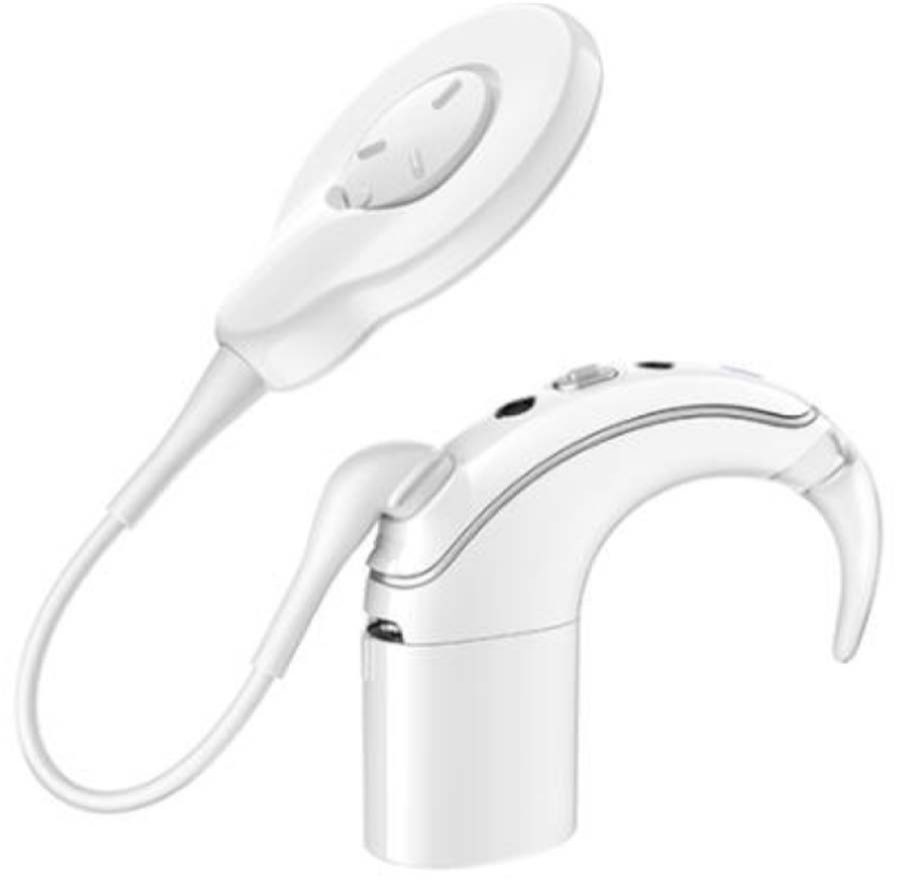
Physical structure of the speech processor.

**Fig 6:**
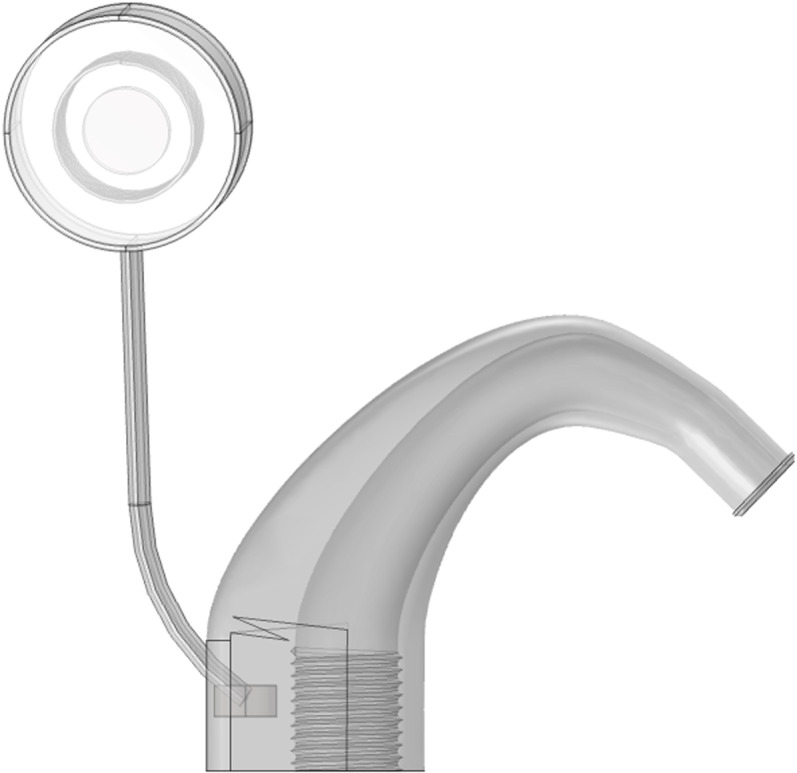
The speech processor model.

Cochlear implant is a recognized method for the treatment of severe bilateral deafness via direct electrical stimulation of the auditory nerve. In this study, as shown in Fig 3(implanter) and Fig 5(extracorporeal part), the cochlear implant is modeled based on the actual size and material of the cochlear implant device, which mainly includes a sound processor located outside the human ear and an internal implant consisting of a receiving processor, magnets and an electrode sequence. Given the complex internal structure of the cochlear implant, this study simplifies some model construction details. For example, only some of the 22 electrodes in the cochlea are retained [31]. The whole cochlear implant model is shown in Fig 4 and Fig 6, and its parameters are given in Table 1.

**Table 1:** Parameters of the cochlear implant model.

Name	Materials or parameters
Cochlear Implant casing	Titanium alloy
Insulating layer	Silicone rubber
Protective outer shell	Silicone elastomer
Coil	Gold
Battery	Lithium
Magnet	Samarium Cobalt
Electrode conductor	Platinum-iridium alloy
Electrode length	25mm
Electrode diameter	0.3mm
Arrangement mode	Spiral arrangement

### Human model and electromagnetic parameters

**Fig 7:**
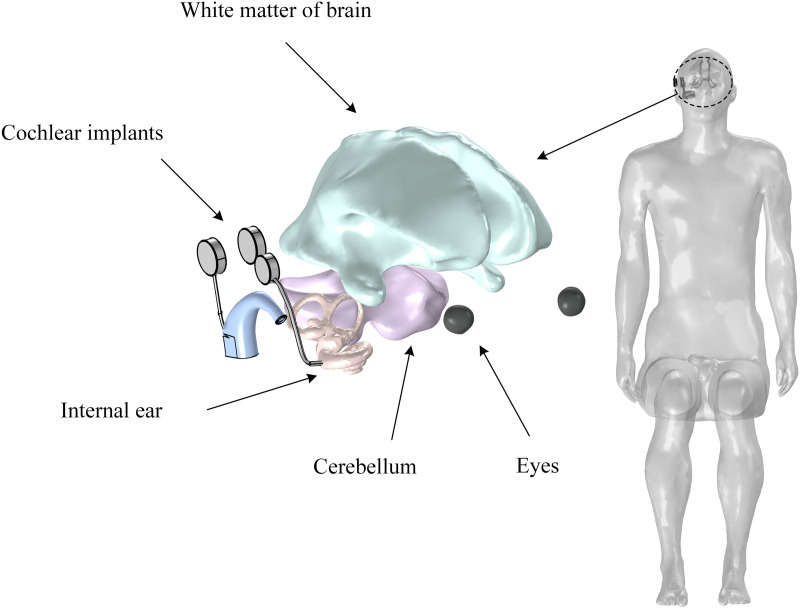
Human model with a cochlear implant.

In this study, an adult male anatomical model [32] based on high-resolution MRI scanning is constructed, and the organs such as white matter, cerebellum, skull, eyeballs, and inner ear, are extracted to construct a sitting model, as shown in Fig 7. The main parameters of human dielectric properties in the calculation are electrical conductivity and dielectric constant. On the basis of the four-order Cole–Cole model [33], this study calculates the dielectric parameters of different human tissues. The dielectric parameters of the human torso are taken as the average values of skin, blood, muscle, and bone tissues, and the dielectric parameters of the inner ear are approximately taken as the reference values of the skull. The thermal parameters related to human temperature rise include tissue density, specific heat capacity, thermal conductivity, and blood perfusion rate. Table 2 shows the relevant electromagnetic parameters of different tissues in the human body.

**Table 2:** Relevant parameters of human tissues (*f* = 80 MHz).

tissue	σ (S/m)	ε	ρ $\left(kg/m^{3}\right)$	$C_{p}$ J/(kg∘C)	k W/(m∘C)
skin	0.4843	76.1495	1109	3391	0.37
blood	1.2196	81.0520	1050	3617	0.52
muscle	0.6977	68.7690	1090	3421	0.49
Bone	0.1783	27.6897	1908	1313	0.32
White Matter	0.30783	61.703	1040	3700	0.50
Skull	0.06183	15.911	—	—	—
Cerebellum	0.75494	101.46	1038	3687	0.57
Eye	1.0183	81.137	1010	4178	0.58

## Analysis of calculation results

### Distribution of electric field intensity in vehicle space

**Fig 8:**
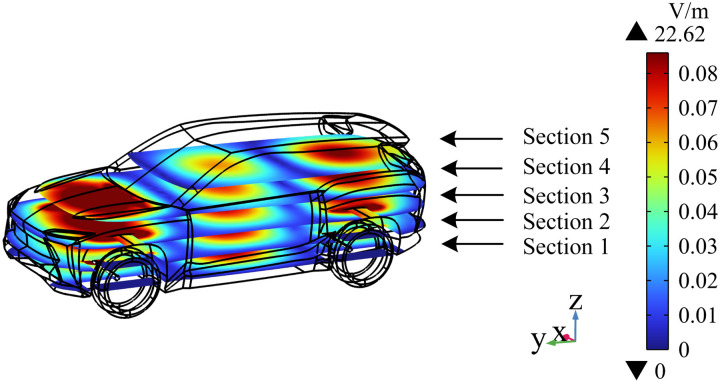
Cross sections of electric field intensity at different positions in the carriage.

Five cross sections parallel to the XY plane are selected to study the distribution of electric field intensity in the EV. As shown in Fig 8, Section 1 is tangential to the center of the radiation source, and the spatial electric field value in this section is large, with a maximum value of 22.62 V/m. When the distance from the radiation source increases gradually, the electric field intensity decreases gradually from Section 1 to Section 5. In addition, the electric field at the front and rear ends of the section is considerably larger than that in the middle region. The electric field intensity in the middle area of the cross section gradually increases from top to bottom because of the location of the radiation source.

### Distribution of induced electric field intensity in the human torso

**Fig 9:**
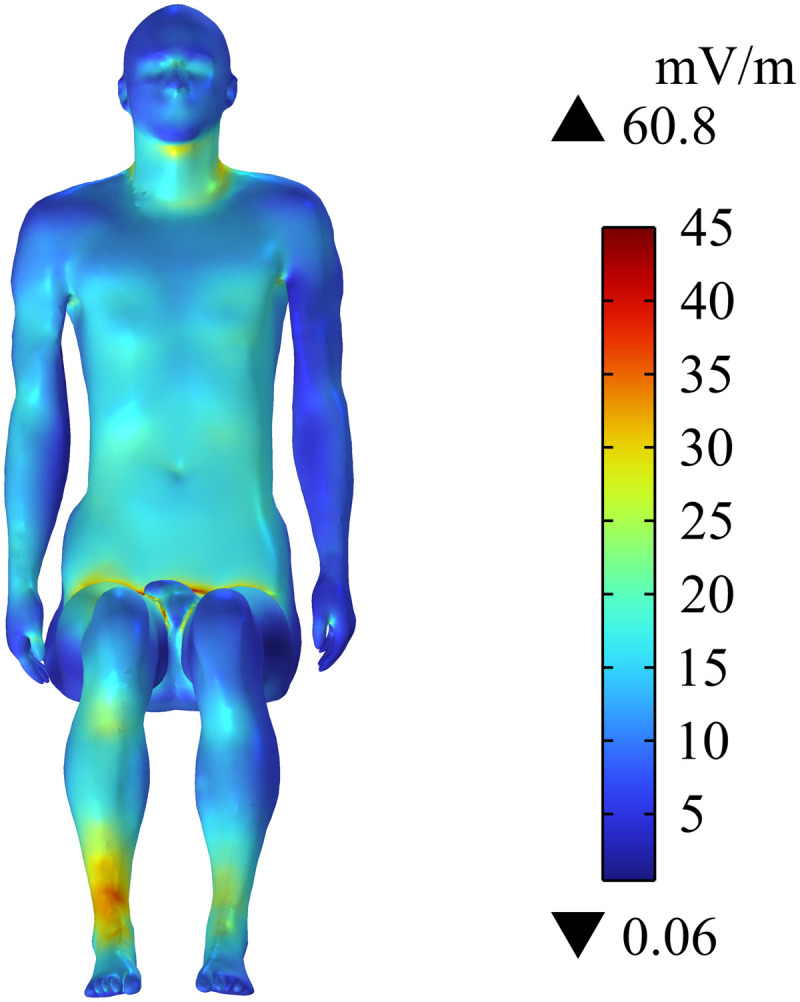
Distribution of $E_{in}$ on the human body surface.

**Fig 10:**
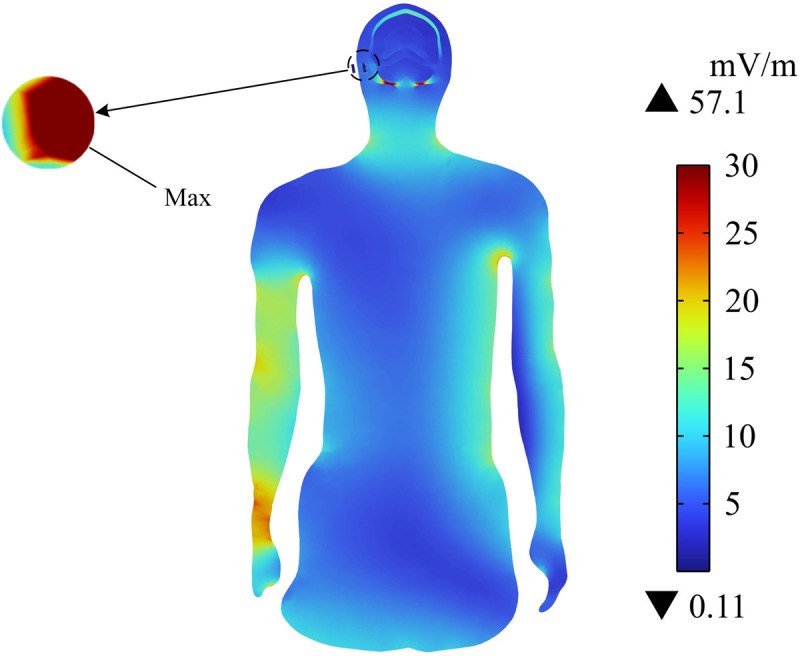
Distribution of $E_{in}$on the central section of the human upper limb.

The distribution of the induced electric field ($E_{in}$) on the human body surface of passengers is shown in Fig 9. The neck, arm, chest, abdomen, calf, and ankle have high $E_{in}$, whereas the other parts of body have relatively low $E_{in}$. $E_{in}\;$at the passenger’s ankle has the maximum value of 60.8 mV/m because $E_{in}$ is inversely proportional to the distance from the radiation source. Fig 10 shows the distribution of $E_{in}$ in the central section of the upper limb. It can be seen that $E_{in}\;$around the cochlear implant area is high, and its maximum value is 57.1 mV/m. Meanwhile, although $E_{in}$ in the passenger’s arm area is also high, it is still lower than $E_{in}$ around the cochlear implant. In addition, $E_{in}$ in the rest of the upper limb is much smaller than at the passenger’s arm. $E_{in}$ on the surface and central section of the passenger’s body does not exceed the public exposure limit (27.7 V/m) for electromagnetic fields specified in ICNIRP 2020. It should be noted that $E_{in}$ near the cochlear implant is considerably high.

### Distribution of induced electric field intensity on the human head

**Fig 11:**
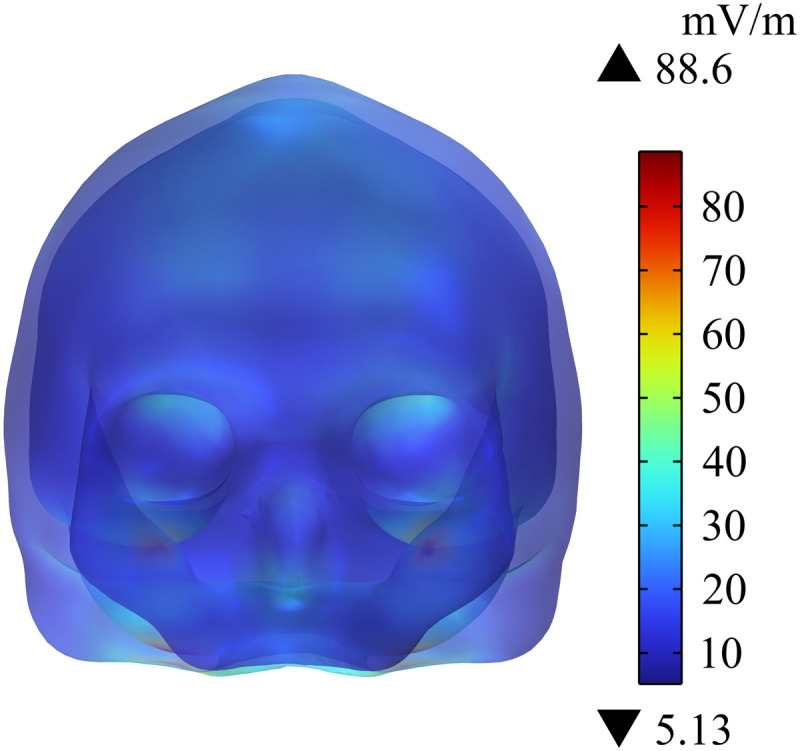
Distribution of $E_{in}$in the skull.

**Fig 12:**
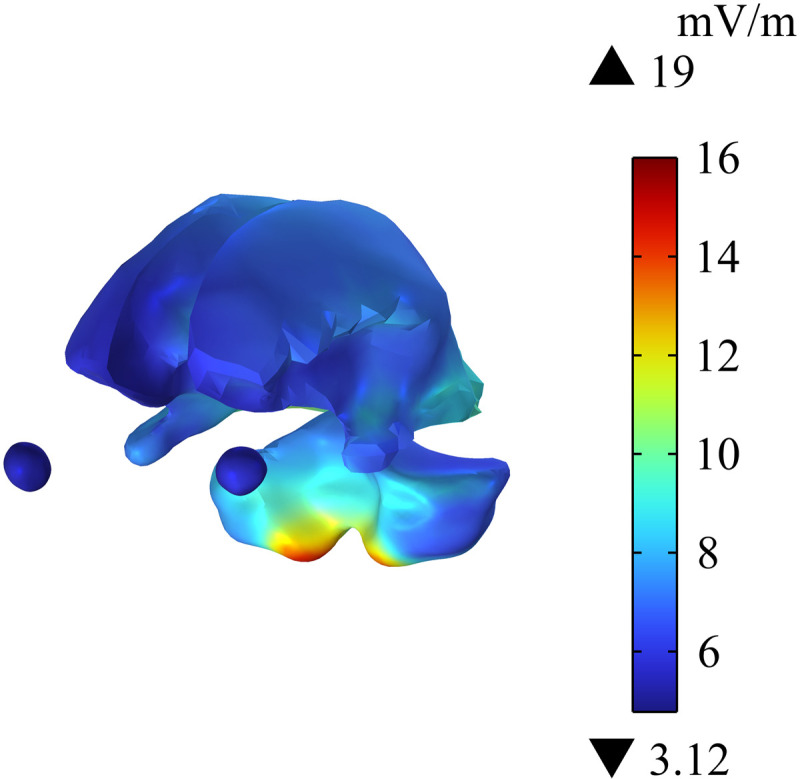
Distribution of $E_{in}$ in white matter, cerebellum and eyeball.

**Fig 13:**
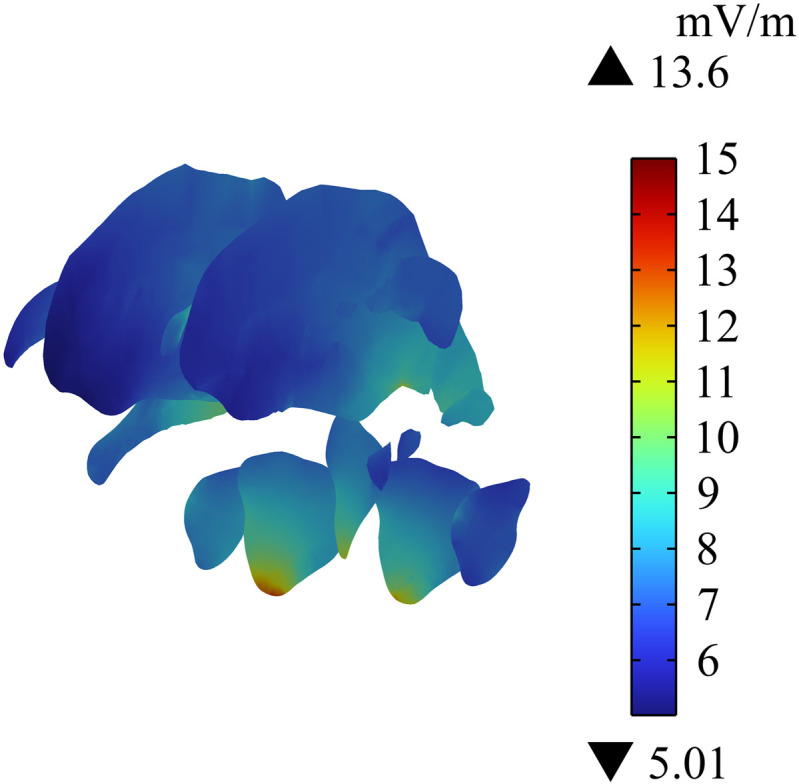
$E_{in}$ in different sections of brain tissue.

Cochlear implant is placed close to human brain tissue. Thus, this study analyzes the distribution of $E_{in}$ in different tissues in the head of passengers wearing cochlear implant. Fig 11 shows that the maximum $E_{in}$ in the head is 88.6 mV/m, and the largest value is at the edge of the skull. Figs 12 and 13 show the distribution of $E_{in}$ in white matter, cerebellum, eyes, and different sections of brain tissues. Compared with $E_{in}$ in the white matter and the eyes, $E_{in}$ in the cerebellum is larger, with a maximum value of 19 mV/m. The different sections of brain tissue reveal that $E_{in}$ in the cerebellum is obviously larger than that in white matter, which is mainly related to the large dielectric constant and high conductivity of the cerebellum. $E_{in}\;$ in the human head tissues is also within the safety limits specified in ICNIRP 2020 guidelines.

### Distribution of SAR on the human torso

**Fig 14:**
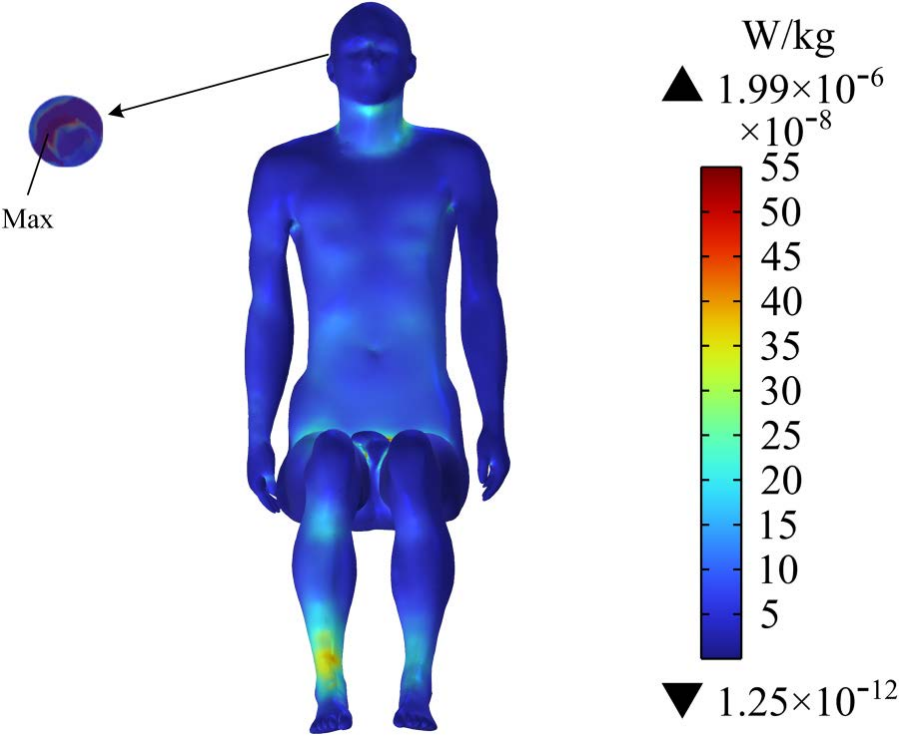
Distribution of SAR on the human body surface.

**Fig 15:**
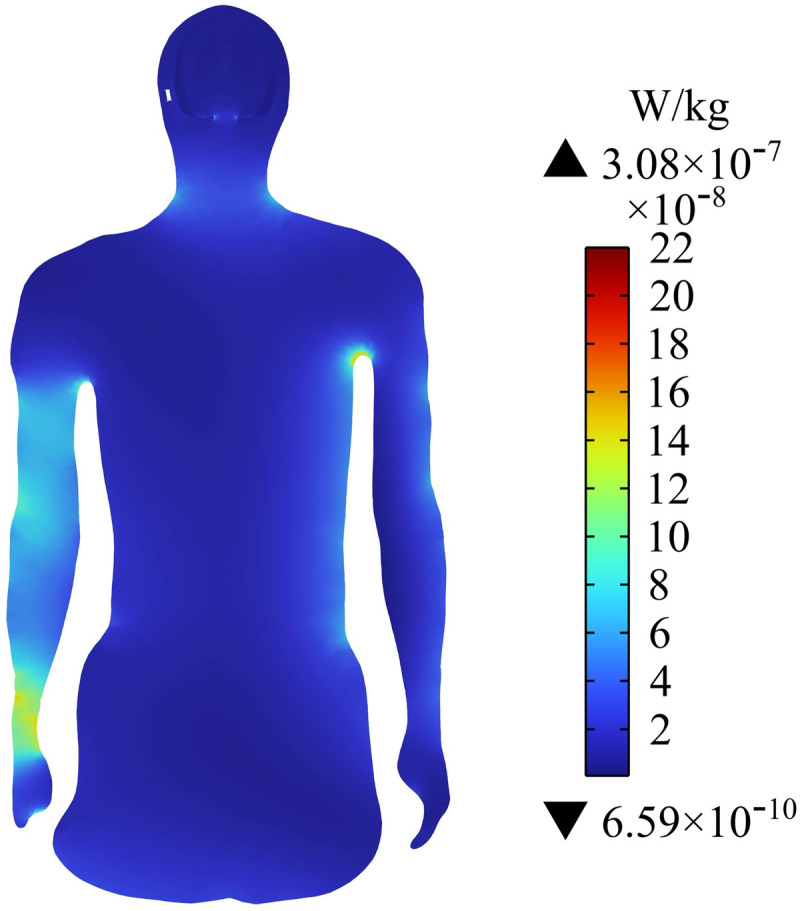
Distribution of SAR in the central cross section of the human upper limb.

Fig 14 shows the distribution of SAR on the passenger’s body surface. The SAR on the neck, arms, and calf are large, and the maximum value is $1.99\times 10^{-6}\;W/kg$ near the cochlear implant of the passenger’s head. Fig 15 shows the SAR in the central section of the upper limb of the human body. The SAR near the arm of the passenger is large, and the maximum SAR in the arm is $3.08\times 10^{-7}\;W/kg$. After calculation, the average SAR of the passenger’s whole body is determined to be 3.02$\times 10^{-8}$ W/kg. ICNIRP 2020 guidelines indicate that the average SAR value must not exceed 8 $\times 10^{-2}$ W/kg, so the SAR of the human body in this exposure scenario is much lower than the exposure limit specified in ICNIRP 2020. However, the SAR near the cochlear implant is much larger than that in the surrounding tissue.

### Distribution of SAR in human head tissues and organs

**Fig 16:**
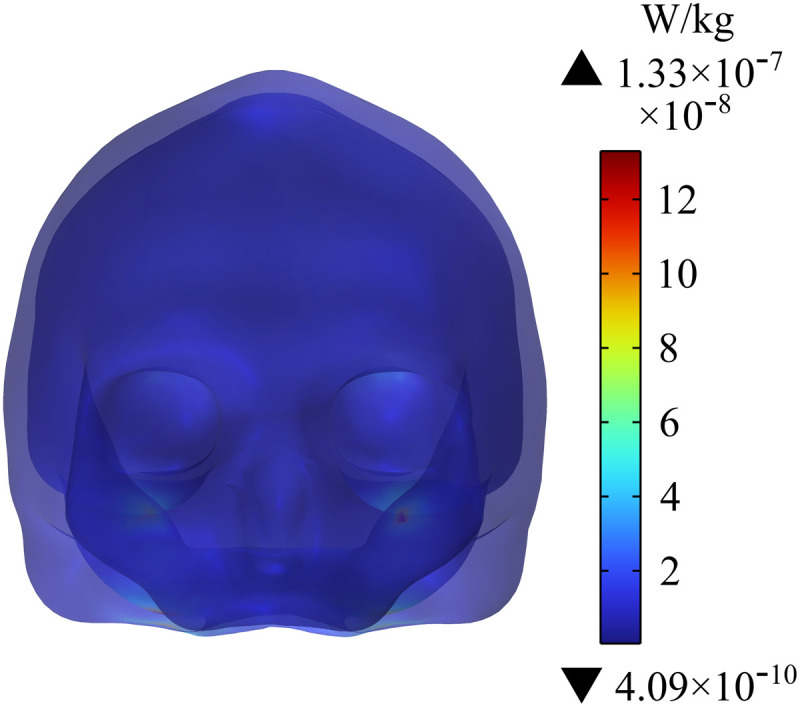
Distribution of SAR in the human skull.

**Fig 17:**
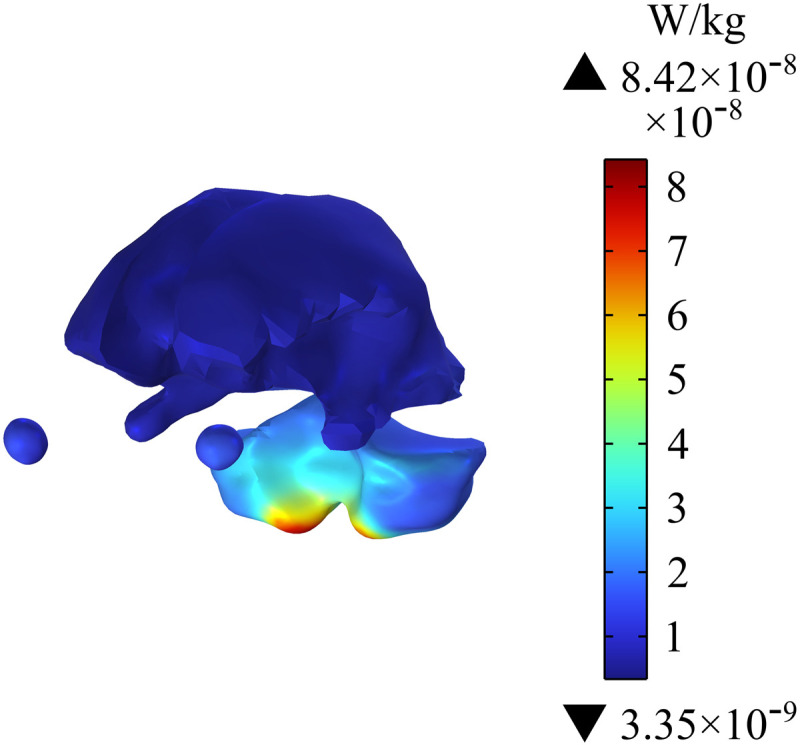
Distribution of SAR in brain tissues.

**Fig 18:**
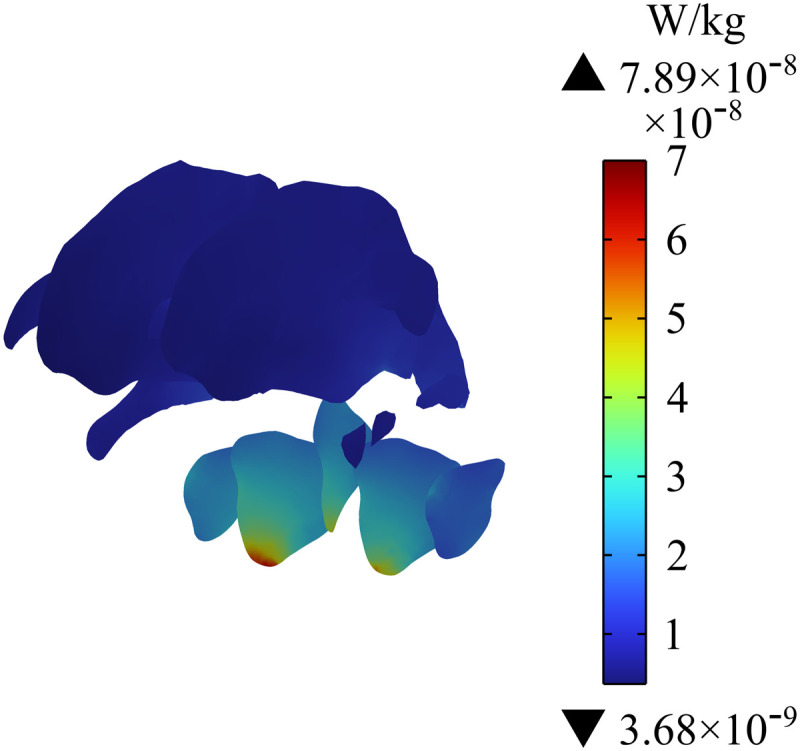
SAR in different sections of brain tissues.

Figs 16 - 18 show the distribution of SAR in the passenger’s head. Fig 16 indicates that the maximum SAR on the head is $1.33\times 10^{-7}\;$W/kg, and the maximum value is at the edge of skull. Fig 17 shows the distribution of SAR in the human brain tissues of the passenger; the maximum SAR with a value of $8.42\times 10^{-8}$ W/kg appears in the lower cerebellum. In addition, the different sections of brain tissues in Fig 18 show that SAR in the cerebellum is much larger than that in white matter. Given that SAR is positively correlated with $E_{in}$, the distribution of SAR in the cerebellum is roughly similar to that of $E_{in}$. Compared with the SAR safety limit specified in ICNIRP 2020, the SAR in human brain tissues in this study is far lower.

### Temperature rise of the human torso

**Fig 19:**
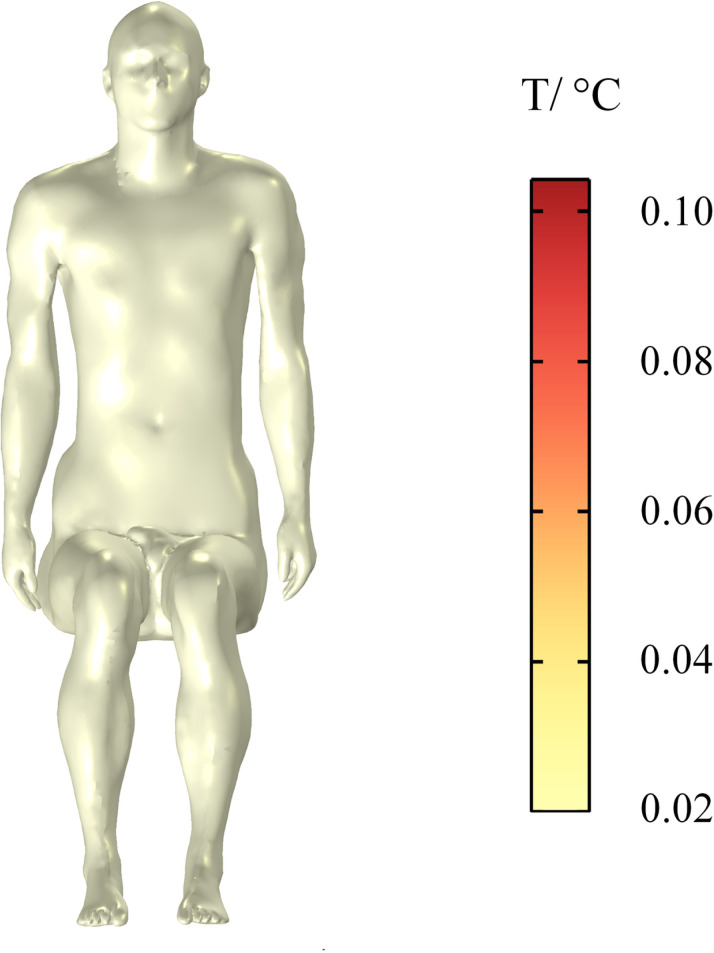
Temperature rise in the human body.

Given that biological tissues convert electric field energy into heat energy after absorbing electromagnetic waves, a biological thermal effect is produced under the action of the cumulative effect. In accordance with the limit of human temperature rise in ICNIRP guidelines, the temperature rise in the human torso, brain tissues, and eyes within 30 min is calculated and compared with the specified temperature rise threshold specified by ICNIRP. In this study, the initial temperature of human tissues is 36.4 °C. The temperature change in the passenger’s torso is shown in Fig 19. The temperature of the whole body increases by a maximum of 0.10 °C within 30 min. The distribution of the temperature rise on the surface of the body is uniform, and the temperature of all parts of the body exhibits a slight increase.

### Temperature rise of the head

**Fig 20:**
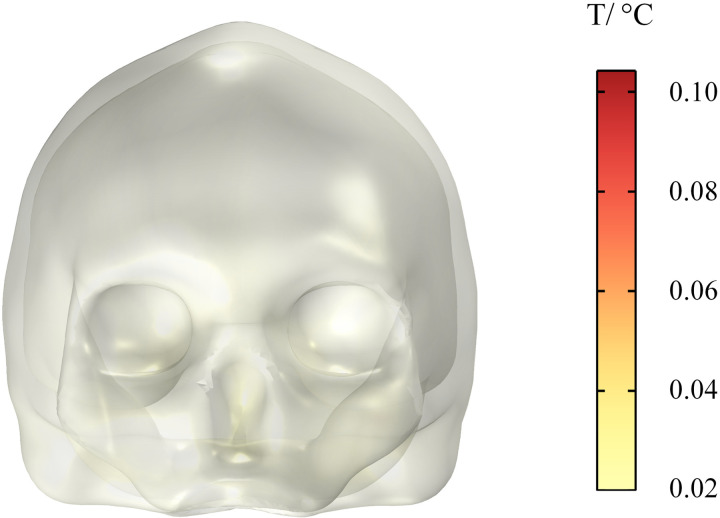
Temperature rise of the human skull.

**Fig 21:**
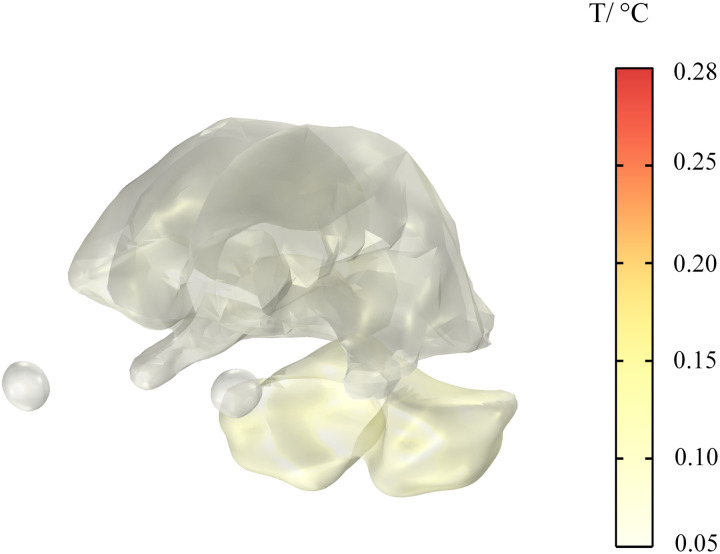
Temperature rise of brain tissues.

As electronic devices, cochlear implant come into direct contact with the nervous system and transmit information to the brain via electrical signals. Their effect on the temperature rise of brain tissue is critical to assessing their safety. The temperature rise of different tissues in the human head are shown in Figs 20 and 21. Within 30 min, the temperatures of the skull, cerebellum, white matter, and eyeballs increase by 0.10 °C, 0.28 °C, 0.17 °C, and 0.0013 °C, respectively. Among these body parts, the cerebellum has the highest temperature rise, but its maximum temperature rise does not exceed the threshold range of heat damage (1 °C) in ICNIRP 2020 guidelines, so it does not affect normal physiological activities of the human body.

### Temperature rise of cochlear implant

**Fig 22:**
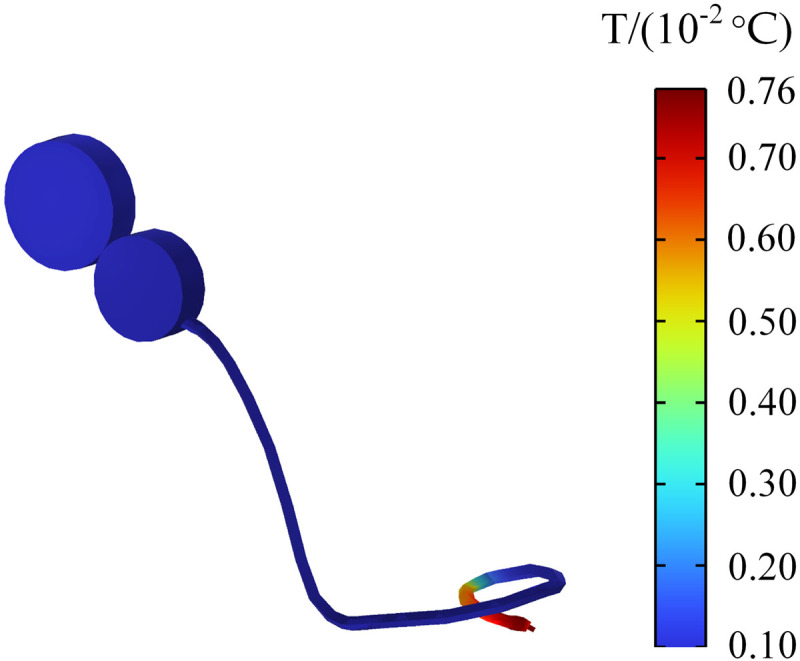
Temperature rise of the cochlear implant.

When a passenger wearing a cochlear implant is in an electromagnetic environment, the electromagnetic field may produce a coupling effect with the electronic components in the cochlear implant. Converting electromagnetic field energy into heat causes the implant to heat up. To ensure passenger safety, this study also calculates the temperature change of cochlear implant within 30 min. Fig 22 indicates that the largest change in cochlear implant temperature occurs at the tip of the cochlear implant electrode, and the maximum temperature rise is 0.0076°C, which meets the requirement of the International Organization for Standardization’s (ISO) 14708–7 standard, which states that the temperature of medical equipment should not exceed 2 °C [34]. Therefore, the electromagnetic environment generated by drive motors does not affect the normal function of cochlear implant.

## Discussion

A cochlear implant is an electronic device used by people with severe hearing impairment to partially restore the hearing function. With the advancing in technology and the increasing in the demand for cochlear implants, these devices have developed tremendously over the past few decades. According to a market research report, the global cochlear implant market reached $1.5 billion in 2020, and the market is expected to reach $3.2 billion by 2028. Cochlear implants have many electronic components, so wearers need to pay special attention to complex electromagnetic environments to ensure safety.

Compared with vehicles with traditional internal combustion engines, EVs have more complex electronic systems and use electric energy as the main power source. During driving, the drive motor, as a core component that converts electrical energy into mechanical energy, generates electromagnetic radiation in its surrounding space. As cochlear implant wearers become more likely to travel in EVs, the electromagnetic radiation may interfere with the normal operation of cochlear implant, thus affecting the hearing function and safety of the wearer.

So far, there has been little study on the impact of the electromagnetic environment in EVs on the electromagnetic exposure of passengers wearing cochlear implants. Therefore, this study analysis the effects of electromagnetic radiation on passenger with cochlear implant during the operation of drive motors to evaluate the safety of EV for this group of people. Specifically, the distribution of the $E_{in\;}$, SAR, and the temperature rise in the different organs and tissues of passenger wearing cochlear implant are calculated at a radiation frequency of 80 MHz. The results are all below the exposure limits specified in ICNIRP 2020 and ISO 14708–7. Considering the complexity of the external electromagnetic environment, in the long run, advanced shielding materials (such as conductive polymers and metallic coatings) should be incorporated into the internal and external components of the cochlear implant when designing the implant. At the same time, for EVs, the electromagnetic shielding capability of the cabin and the optimal layout of the power system should be considered to reduce the electromagnetic field exposure level inside the cabin.

## Conclusion

Since cochlear implant users often have severe hearing impairment, their health and safety are particularly important in traffic environments. By analyzing the effect of the electromagnetic environment generated by drive motors of EV on passenger with cochlear implant, this study finds that although cochlear implant affects the distribution of $E_{in\;}$ and SAR in the local area of passengers’ head, the electromagnetic radiation generated by the drive motors does not affect the safety of passenger with cochlear implant and does not pose a threat to the normal function of the implant. These findings not only enrich the study on the electromagnetic environment of EVs, but also provide reassurance to cochlear implant users and guide future advancements in implant design and vehicle safety standards.
